# Chirality-Dependent Adsorption between Amphipathic Peptide and POPC Membrane

**DOI:** 10.3390/ijms20194760

**Published:** 2019-09-25

**Authors:** Ke Chen, Yuebiao Sheng, Jun Wang, Wei Wang

**Affiliations:** National Laboratory of Solid State Microstructure, Collaborative Innovation Center of Advanced Microstructures, and School of Physics, Nanjing University, Nanjing 210093, China; chenchk012@163.com

**Keywords:** chirality, protein, membrane, POPC, tryptophan, molecular dynamics

## Abstract

The interactions between chiral molecules and cell membranes have attracted more and more attention in recent decades, due to their importance in molecular science and medical applications. It is observed that some peptides composed of different chiral amino acids may have distinct interactions with a membrane. How does the membrane exhibit a selective behavior related to the chirality of the peptides? Microscopically, the interactions between the peptides and the membrane are poorly understood. In this work, we study the interactions between an amphipathic peptide (C6) and POPC membrane with simulations. The kinetics and thermodynamics of peptide enantiomers during the adsorption to the membrane are characterized with direct simulations and umbrella sampling. It is observed that there are slow kinetics for the peptide composed of D-type amino acids. Along the observed pathways, the free energy landscapes are determined with umbrella sampling techniques. A free-energy barrier for the peptide composed of D-amino acids is observed, which is consistent with the kinetic observations. The results indicate the concurrent adsorption and rotation of the peptide helix. The local interactions between the peptides and the membrane are examined in detail, including the contact interactions between the peptides and the membrane, and the distributions of the lipids around the peptide. There are observable differences of the local interactions for the cases related to different peptide enantiomers. These results further demonstrate the importance of the rotation of peptide helix during the adsorption. More interestingly, all these kinetic differences between peptide enantiomers can be explained based on the conformations of the residue Trp and interactions between Trp and lipid molecules. These results give us a molecular understanding of the mechanism of the chirality-dependent peptide–membrane interactions, and may provide clues to designing systems which are sensitive to the chirality of membranes.

## 1. Introduction

Chirality is a property of many multi-atom molecules whose mirror objects are different from themselves. Emergence of chirality is often attributed to the presence of asymmetric carbon atoms, as those in chiral amino acids and lipids as well as the proteins and membranes which are composed of the chiral units. This kind of local asymmetry produces different geometries of the molecules, and affect the interactions and functions between the chiral molecules. For example, specific chirality is required in many enzymatic catalyses and degradations due to geometric restrictions [1,2,3]. Variation in the chirality of the molecules would weaken or destroy their functions. To understand the role of chirality in molecular interactions is one of many important topics in molecular science [4].

Among various biomolecular systems, membranes have been extensively studied by experiments and computational models [5,6,7,8,9]. The chirality of membranes is often overlooked. Since chiral lipid molecules are rather flexible, the membrane is often modeled in a mean-field manner as a medium with complex composition [10,11]. Based on these models, some successes were achieved to characterize the interactions between various molecules and membranes [12,13]. Nevertheless, there are an increasing number of experiments illustrating the selective interactions between membranes and various ligands. It is reported that membranes can discriminate enantiomers of phenylproline tetrapeptides in passive diffusion through the blood–brain barrier [14]. The adsorption of ibuprofen to liposome membranes was also found to be chirality-dependent [15]. A similar chirality-dependent effect is also found for bilirubin [16,17] and biliverdin [17]. More importantly, chirality-related interactions between membranes and natural amino acids (in monomeric/dimeric forms) are also observed [18,19,20,21,22,23,24,25]. In detail, it was reported that L-type amino acids, compared to the D-type ones, were more favored to be adsorbed to DPPC liposomes [18]. Among various natural amino acids, the tryptophan (W, Trp) has the most remarkable difference between its enantiomers [18]. Note that all the dependencies involve no specific receptors in the membrane (receptor-free), but are fundamental aspects imprinted by the intrinsic chirality of membrane lipids and the dynamic self-assembly nature of membranes [15,18,26]. This implies that there are possible chiral interactions between membranes and proteins, which are frequent and important in biological systems. Recently, different interactions between POPC membranes and the enantiomers of a peptide (C6) are observed. This peptide is a designed cell-penetrating peptide analogous to cationic -helical antimicrobial peptides (AMPs) [27]. It is positively charged by +7 e at neutral pH and can fold into amphipathic -helix in both solution and POPC membranes [28]. It is observed that the aggregations of C6 enantiomers in POPC membranes are different in their sizes, densities and lifetimes. In consequence, these enantiomers exhibit different abilities to penetrate POPC bilayers and produce different antimicrobial behaviors [28]. These kinds of chirality-related behaviors are also observed in other peptide–membrane systems [29,30,31]. These events cannot be explained by the medium model of the membrane since the uniform media cannot have the selectivity related to chirality [32], and can only be attributed to the chirality of the lipids in the membrane [31]. It is noted that there are many experiments reporting that the enantiomers of some peptides have no differences in their functions when they interact with membranes [3,33,34,35,36,37,38,39,40]. Clearly, not all enantiomers of peptides or proteins interact with membranes differently. The contradictive results indicate the importance of the peptide sequences. It is also observed that changes in membrane composition can lead to diverse responses to small chiral molecules [15] and peptides [29]. All of these facts further emphasize the subtlety of the chirality-dependent interactions which is sensitive to both peptide and membrane [29,30,31]. Yet, little is known about the determinants during the interactions between peptides and membranes. To find out when and how the chirality produces significant thermodynamic and kinetic effects during the interactions between membranes and proteins would be valuable for molecular biophysics and drug design.

Indeed, to disclose the physics underlying the chirality-related interactions between peptides and membranes is a difficult task in present stage, since the attraction between the peptide and the membrane is dominant during various processes (such as adsorption, insertion and so on), while the chirality-related interactions are rather marginal and produce subtle observations. Thus, the selectivity to enantiomers are sensitive to the composition of peptides and membranes. For the peptide enantiomers evidently discriminated by membrane, mixed composition of membrane is often required [29,30]. However, the present experiments are still rare to conclude what kinds of composition of peptide and membrane are the key factors during the interactions. Moreover, some experiments are carried out at the cell level. Some biological biases such as the protease-resistance of peptides with D-amino acids may interfere with the results [3,41,42]. The model systems with relative simple composition and structures are expected. Fortunately, the system with peptide C6 and POPC membrane is the one with simple amino acid composition (only three kinds of amino acids) and lipid composition (with pure POPC bilayer). With this kind of composition, the peptide chain has relatively strong preferences to form α-helix, both in aqueous environment and in the membrane [28]. Based on these considerations, the system with the enantiomers of C6 peptide in POPC membrane is employed as the model system in our simulations. We believe that this model system may help us to discover the determinants during chirality-related peptide–membrane interactions.

In this work, we focus on the interaction between the POPC membrane and the enantiomers of the amphipathic peptide C6. The adsorption process of a peptide on the membrane is simulated. It is observed that C6 enantiomers have different kinetics. The C6 peptide composed of L-type amino acids (named as LC6) is generally adsorbed fast, while the one with D-type amino acids (named as DC6) may be adsorbed rather slowly. Following the selected adsorption pathways for these enantiomers, umbrella sampling simulations are carried out. The free-energy landscapes are determined. The results indicate that peptide LC6 has a single funnel and is adsorbed in a downhill manner, while the peptide DC6 is adsorbed with a free-energy barrier which results in a slow kinetics. Through detailed structural analysis, it is found that the barrier crossing process for peptide DC6 is accompanied by a directional rotation along its helix axis. The kinetic differences between these two enantiomers are attributed to the differences of contacts and lipid distributions around the peptides. More interestingly, the downhill and barrier-crossing pathways can be distinguished from each other by the orientation of tryptophan and the preferential interactions between tryptophan and lipid head groups. We believe that our study may provide a better understanding of chirality-related interactions between membranes and peptides.

## 2. Results and Discussion

### 2.1. Why Adsorption of the Peptide Is Interested

**Figure 1:**
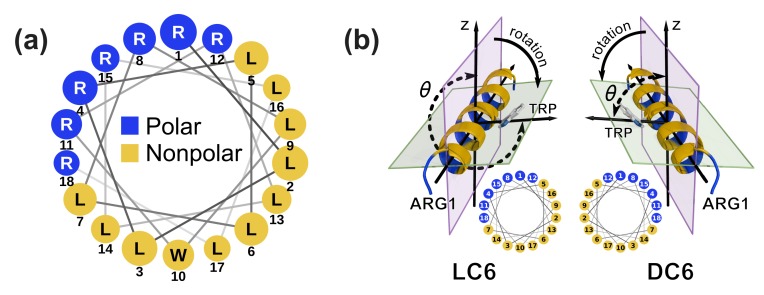
(**a**) Wheel projection of peptide LC6. The sizes of the residue labels (circles) are scaled according to the location of the residue along the helical axis (larger at the N-terminus and smaller at the C-terminus). DC6 is the enantiomer of LC6. (**b**) Diagram of angle θ which characterizes the rotation of C6. The residues are colored in the same manner as in the wheel projection (blue for polar residues and yellow for nonpolar residues). The rotation angle θ is measured with the dihedral composed of the axis of the helix, the side chain of Trp (atom CG) and the positive direction of z-axis. See Materials and Methods for further elaborations. The arrows marked by “rotation” denote the rotation directions of C6 enantiomers. Small wheel projections of the enantiomers are shown to demonstrate the mirror symmetry. The wheel projection is plotted with NetWheels [51].

Peptide C6 (with the sequence Ac-RLLRLLLRLWRRLLRLLR-NH${}_{2}$) is an analogue to cationic -helical AMP. It adopts a helical structure with amphipathic feature, with the charged and the hydrophobic residues distributed on two sides of the helix (as shown by the wheel projection for LC6 in Figure 1a, and DC6 is the mirror image of LC6). The helical structure is stable in aqueous environment and in the membrane for both LC6 and DC6 (as indicated by the CD spectra in experiments [28]). On the basis of the composition and the structure, peptide C6 is similar to the AMP melittin [3] in the feature of amphipathicity and a large number of charged residues (+7e for C6 [27] and +5e for melittin at neutral pH condition). According to the classification of antimicrobial peptides [43,44], it is reasonable to postulate that peptide C6 behaves similarly to melittin during the interaction with the membrane. On the basis of the studies on melittin [45,46,47,48], the adsorption process is a prerequisite to realize the pore-formation function that may lead to membrane penetration and cell lysis [45]. Therefore, to study the adsorption of peptide C6 is a valuable step to understand peptide–membrane interactions. More importantly, based on the knowledge about melittin, the adsorption of AMP helix would experience a dive into the boundary region between the head and tail groups of the lipids, as well as a rotation of the helix along its axis (as denoted in Figure 1b and characterized by the angle θ) [49,50]. Since the chiral carbon atom of the lipid molecule is in this region, the interaction between the peptide and the chiral carbon atoms of lipids may be frequent during this process. This may help us to understand how chirality affects the interactions between the membrane and the peptide. Additionally, in peptide C6, there is a tryptophan residue, whose monomeric enantiomers have large differences during the adsorption to the membrane [18]. The existence of this amino acid may be another reason or indicator to explain the differences between the enantiomers of peptide C6 when they are interacting with the membrane. These may provide clues to elucidate the functional variance of C6 enantiomers observed in experiments.

### 2.2. Adsorption of Monomeric Peptide C6

**Figure 2:**
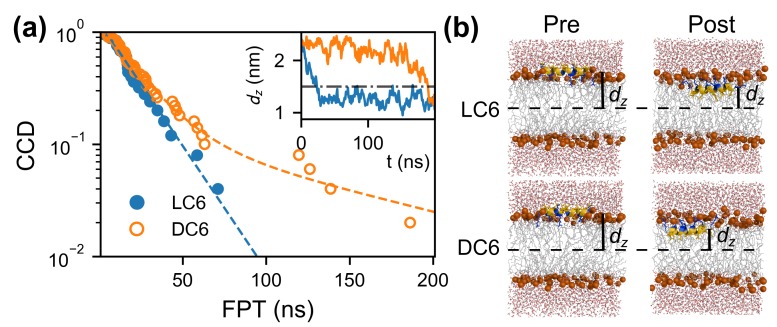
(**a**) The CCD (circles) and exponential fittings (dashed lines) of FPT at $d_{z}=1.5\;\mathrm{nm}$. The CCD of DC6 is fitted with a hybrid of two exponential distributions with normalized weights. $d_{z}$ records for typical trajectories are shown in the inset cell. The dot-dashed line indicates the location of $d_{z}=1.5\;\mathrm{nm}$. (**b**) The typical pre-adsorption and post-adsorption snapshots of monomeric simulations. Coloring of the peptide follows the scheme in Figure 1. The phosphorus of the membrane are shown as orange spheres. The lipid tails are shown in gray and those in the foreground of the peptides are removed for clarity.

To investigate the adsorption of peptide C6, we carry out a series of constant-temperature simulations (25 trajectories for LC6 and 50 ones for DC6) for the adsorption process of the monomeric peptide. During these simulations, the first passage time (FPT) is defined as the time when the center of mass (COM) of the peptide for the first time passes the location $d_{z}=1.5nm$, where $d_{z}$ is the z component of distance between the COMs of peptide and of the membrane bilayer, as shown in Figure 2b. This definition of FPT corresponds to the achievement of the adsorption. The typical initial and final configurations of adsorptions are shown in Figure 2b. These configurations are named respectively as the pre-adsorption and the post-adsorption states, which are defined quantitatively later. For all these kinetic trajectories, the FPTs are collected. Based on these data, the complementary cumulative distributions (CCD) of FPT are determined as shown in Figure 2a. Two kinds of kinetics for C6 enantiomers are observed. For peptide LC6, the CCD decays exponentially with a unique time constant of 19.61+-1.07 ns. Here, the exponential distribution suggests that all the LC6 peptides follow an identical kinetics. Indeed, a better fit for this CCD function can be achieved with a gamma distribution. This indicates that the adsorption of LC6 peptide might be in a downhill manner. Differently, for peptide DC6, the CCD function has a long tail, deviating far away from the initial exponential decay. This tail indicates that some adsorption processes are comparatively slower comparing to the average cases. As the orange trajectory shown in the inset of Figure 2a, the peptide fluctuates above the membrane for a long time (larger than 100 ns), and finishes the adsorption quickly after that. This is likely to be an activation process. Practically, this CCD function is fitted by a function composed of two exponential terms which correspond to fast and slow parts of the kinetics. During the fitting, the weights applied to these two terms are always normalized. The time constants for these two terms (i.e., the two kinds of kinetics) are 19.59+-2.70 ns and 117.4+-53.0 ns, respectively. The time constant of the fast mode is very close to that of LC6, suggesting that the peptide DC6 (in fast mode) and LC6 have similar kinetics. That is to say, there are no contributions from the chirality of the molecules. The slow adsorption kinetics is particular for the DC6 peptide, and the fraction (weight of the corresponding term) is about 10%. Moving with this kind of slow kinetics, multiple DC6 peptides may be blocked with improper locations and/or orientations. In consequence, these peptides may form large aggregations above (rather than penetrate into) the membrane, and thus deteriorate the antimicrobial function. Coincidentally, this ratio is very close to the difference of the leakage percentage between LC6 and DC6 at the same peptide concentration [28]. On the basis of such fraction, the fast mode is alternatively mentioned as the normal kinetics of DC6 hereafter. It is worth noting that the only difference between the peptides LC6 and DC6 is the chirality of the residues. The slow kinetics should be a result of the chirality-related interactions. Given the mirror symmetry of enantiomers, there must be other asymmetric factors to induce such kind of kinetic anomaly, since uniform media cannot respond differently to the peptide enantiomers. Considering that the lipids in membrane move freely, the interactions between lipids cannot produce the chirality-related interactions during the long time scale related to peptide adsorption/penetration and further functional behaviors. In this simple system, the only reason would be the chirality of the lipid molecules. How does the chirality produce such kind of kinetics would be the central question in our work.

### 2.3. Umbrella Sampling on Typical Pathways

According to the above simulations, the chirality of the peptide DC6 produces a special slow kinetics, which is related to the chirality-related interaction. To investigate these slow kinetics can help to disclose how the chirality affects the interactions. We carry out the umbrella sampling along the slow pathway to investigate the conformational variations and interaction characteristics during such processes. This is a typical method to study the adsorption and binding processes [50,52], and can be used to evaluate thermodynamic features and detailed microscopic information of the concerned systems. As a comparison, the adsorption for peptide LC6 is also investigated with the same method.

**Figure 3:**
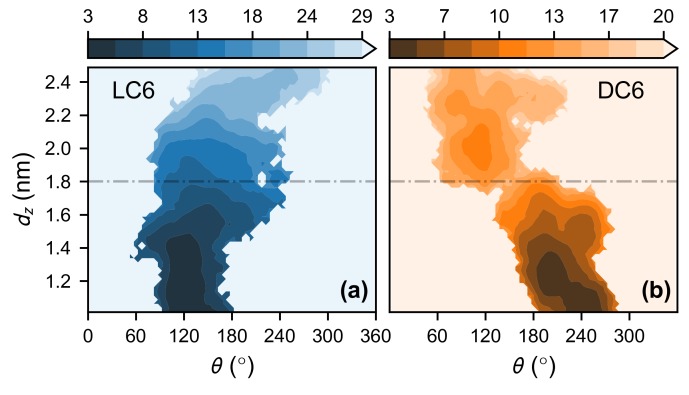
The PMF landscapes for the adsorption of LC6 (**a**) and of DC6 in the slow mode (**b**). The landscapes are reweighted from the umbrella sampling simulations. The PMF values are in $k_{B}T$ with T=303.15K.

With the umbrella sampling techniques, the landscapes of potential of mean force (PMF) for the peptides LC6 and DC6 are determined (as shown in Figure 3a,b). The location of COM $d_{z}$ and the orientational angle θ (as shown in Figure 1b) are used as the coordinates to describe the relative organization of the membrane and the peptide. The stability of the adsorbed state makes the landscape being funnel-like. This is true for both enantiomers, and forms the background of the chirality-related interactions.

Clearly, during the adsorption, the rotations are involved for all the peptides. This is the requirement of the complementarity of interactions between the membrane and the peptide. When the peptide is above the membrane, the interactions between the charged residues and the head groups of lipids make the hydrophobic part of the helix face upward, as shown in the Pre column in Figure 2b. After the immersion into the membrane, the relative position between the peptide and the membrane changes. The charged part of helix has to point up to interact with the head groups properly. At the same time, the hydrophobic part of the helix goes downward and interacts with the nonpolar tails of the lipids, which also helps the decrease of the free energy (the Post column in Figure 2b). Consequently, a rotation is accompanied during the adsorption, as observed in other AMPs [49,50]. The peptide LC6 rotates clockwise following the decrease of the angle θ, and the peptide DC6 moves in an anti-clockwise manner (Figure 1b, and see Figure S1 for detailed elaboration of the rotation). This is originated from the chiral difference between these peptides. The different rotating directions are natural results of the mirror symmetry of enantiomers.

Besides this overall shape of the landscape, there are some other differences between the landscapes of these peptides. For peptide LC6, following the adsorption (namely, the decrease of the center location $d_{z}$), the free energy decreases monotonically. A single funnel is observed. At the starting stage, there is a clear variation of the angle θ, and finally, the angle reaches the value corresponding to the adsorbed state. This is similar to the behavior of the AMP melittin [50]. After that, the adsorption is straight-forward, and the final stable conformation can be achieved. On the other hand, for the peptide DC6, there is a bottleneck on the landscape, around $d_{z}=1.8nm$ and $\theta =150{}^{\circ }$, which separates the landscape into two parts. Following the progress of the adsorption (namely, the decrease of $d_{z}$), a free energy barrier is experienced, though the barrier is marginal (4.3 $k_{B}T$). With this barrier, an activation kinetics is expected and the kinetics would be retarded, which is consistent with the above simulations on the adsorption kinetics of peptide DC6.

Note that the free-energy barrier for peptide DC6 is accompanied with the rotation of the peptide. Two parts of landscapes have distinct averages of the orientational angle (θ). It suggests that the translational motion of the helix is coupled with the rotation of the helix. The emergence of the barrier would be related to the coupling between these two kinds of motions. That is, the chirality-related interactions would happen at a certain position and orientation of the peptide. Both requirements reflect again the subtlety of the chirality-related interactions. This is not observed in the motion of LC6. It indicates that the delicate coupling is broken in the LC6 case. Through this comparison, we could postulate that the chirality-related interaction probably happens locally in some places of the peptide so that the kinetic modes are sensitive to the local geometry of molecules.

### 2.4. Differences in Contact Interactions During the Adsorption of C6 Enantiomers

To understand the microscopic characteristics of the complicated adsorption processes, we need to analyze the local interactions between the peptide and the surrounding lipids. Considering the importance of directed rotations during the adsorption, the helix is likely to be considered as a rigid body (a cylinder), rather than a mass point. Thus, it is necessary to analyze the interactions with spatial resoluton because the forces acting on different parts of the peptide helix will produce different effects. In detail, during the adsorption, the cylinder of the helix rotates around the helix axis along the direction from ARG1 to ARG18, as shown in Figure 1b. During the rotation, one side of the cylinder moves downward, namely, the velocities of the surface residues pointing along the negative *z* axis. This part of the cylinder is defined as the descending part. Meanwhile, the other side of the cylinder moves in an opposite direction, and is correspondingly defined as the ascending part. Clearly, during the adsorption, two parts of the helix interact with the membrane in different styles. The descending part would dive into the membrane from the aqueous environment, and thus compress the membrane, while the ascending part would move in an opposite direction and drag the lipids out of the membrane. These two parts contribute differently to the adsorption process.

**Figure 4:**
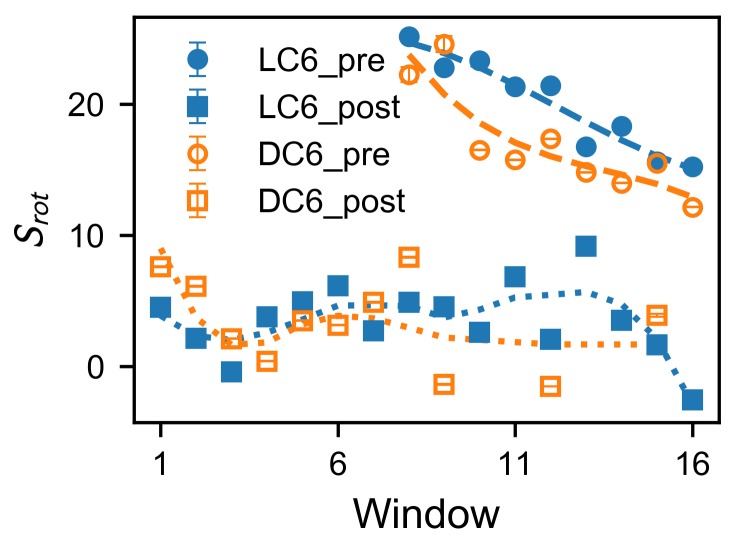
Averaged score for rotation of pre- and post-adsorption states. The standard error of the mean (SEM) is plotted as an error bar. The SEM is a measure of the dispersion of sample means around the true mean and is estimated as $\sigma_{S_{rot}}\approx s/\sqrt{n}$, where *s* is the standard deviation of the sample, and *n* is the size of the sample (number of observed frames). The size of sample for each state in each window is typically 800 to 15,000. Relatively larger SEMs are observed for pre-adsorption states in window 8 and 9 of DC6 due to the relatively smaller sample sizes. Every pair of samples of the pre-adsorption state in each window have different means at the significance level of 0.1, according to Welch’s *t*-test. Guidelines are plotted as signs of the trends.

Geometrically, the lipid–peptide interactions are classified into two parts, i.e., the interactions of lipids with the peptide atoms in the descending and ascending parts. Besides the differences from the geometry of the peptide cylinder, the amino acid composition is another source of the complexity of these interactions. The interactions between lipids and peptide are not uniform, considering that different types of amino acids as well as the head or tail groups of lipid molecules may be involved. As a semi-quantitative estimation, the concerned compositions are coarse-grained as hydrophobic and polar parts of the amphipathic surface of helix. Correspondingly, the interactions are simplified based on the compositions. Clearly, two kinds of interactions, i.e., the hydrophobic interactions between the nonpolar residues and lipid tails, and the polar interactions between charged residues and the head groups of lipids take the important role. Based on these considerations, a weighted score $S_{rot}$ based on local contacts between the peptide and the surrounding lipids is defined as

(1) $$S_{rot}=w_{dp}N_{dp}+w_{dh}N_{dh}+w_{ah}N_{ah}+w_{ap}N_{ap}\;,$$

in which $w_{x}$ and $N_{x}$ are the weight and the number of contacts for the type *x*, and *x* is represented with two symbols ηξ (where η takes *d* or *a*, indicating the descending or ascending part, and ξ uses *h* or *p* giving the type of interaction, hydrophobic or polar). Here, the weight $w_{x}$ describes the contribution of the concerned *x*-type interactions. The determination of the weights as well as their values are given in Supplementary Materials (Table S1, Figure S1 and corresponding paragraphs). The scores $S_{rot}$ are averaged for each window of the umbrella sampling (Figure 4). Clearly, the score $S_{rot}$ considers compositional and structural features of the peptide–membrane system, and measures the interactions between helix and membrane during the adsorption. Note that all the interactions are described as the contacts based on the fact that they are generally short-range interactions.

To compare the variation of interactions during the adsorption, the $S_{rot}$ score is evaluated for both pre-adsorption and post-adsorption states (as shown in Figure 4). These states are exemplified in Figure 2b, and are practically defined here. The post-adsorption state includes the configurations with stable interactions between membrane and peptide, and corresponds to the global minimum in the free-energy landscape in Figure 3. It can be regarded as the fully adsorbed state. Practically, this state is defined as the region $\theta <180{}^{\circ }$ for peptide LC6 (or $\theta >180{}^{\circ }$ fo the peptide DC6) on the free-energy landscape. The pre-adsorption state is another extreme, with the angle θ being far away from the range for the fully adsorbed state. This state describes the configurations where the peptide is not sufficiently immersed in the membrane, and is defined as the region $\theta >225{}^{\circ }$ for peptide LC6 (or $\theta <135{}^{\circ }$ for the peptide DC6). For simplicity, these two states are briefed as “pre” (the pre-adsorption state) and “post” (the post-adsorption state), respectively. Here, the conditions for these states in LC6 and DC6 cases satisfy the chiral symmetry, namely, they can be transformed to each other based on parity transformation. Note that not all conformations are included in the above analysis. The comparison between these two states can remarkably illustrate the difference of interactions before and after the adsorption.

As shown in Figure 4, in the post-adsorption state, these two peptides are similar. Their scores are relatively small. This indicates that contact biases between two sides of the helix are small, which is consistent with the picture that the post-adsorption state (fully adsorbed state) is in the thermodynamic equilibrium. Differently, in the pre-adsorption state, score $S_{rot}$ increases when the peptide approaches the membrane (windows from 16–8, whose center locations $d_{z}$ decreases from 2.5 to 1.7 nm). This is driven by the binding energy between membrane and peptide. During this stage, scores $S_{rot}$ for peptide LC6 are larger than those for DC6. This indicates a large bias to the rotation for peptide LC6, and suggests that there may be stronger interactions in the descending side of peptide LC6. Especially, in windows 12–8, (where $d_{z}$ varies from 2.1 to 1.7 nm), the score $S_{rot}$ for LC6 soon reaches a high level (larger than 20) and keeps increasing, while that for DC6 starts from a lower value 15 and does not reach 20 until window 8 or 9 (corresponding to $d_{z}=1.7$ or 1.8 nm). The fast raise of score for DC6 is consistent with the location of the free-energy barrier. These results imply that a large score $S_{rot}$ may help to overcome the local interactions which impede rotations, while a weaker driving force (such as for DC6) would make the rotational barrier observable. All this information suggest that the different behaviors of peptides LC6 and DC6 come from the pathways of adsorptions, rather than the adsorbed configuration (or final stability). This view is supported by the above kinetic observations.

### 2.5. Nonuniform Distribution of Lipids around the Peptide

The above results demonstrate that membrane–peptide interactions can be different for C6 enantiomers. This cannot be explained by the medium model, and the molecular details are necessary to understand the phenomena. These results also provide some clues for the study of the membrane–peptide interactions. Since the driving force for the rotation is phenomenologically essential for certain kinetic modes, the interactions at the descending and ascending sides would be important because they are responsible for the variation of driving force. Focusing on these certain sides of the peptides may bring us more information about the chirality-related interactions.

**Figure 5:**
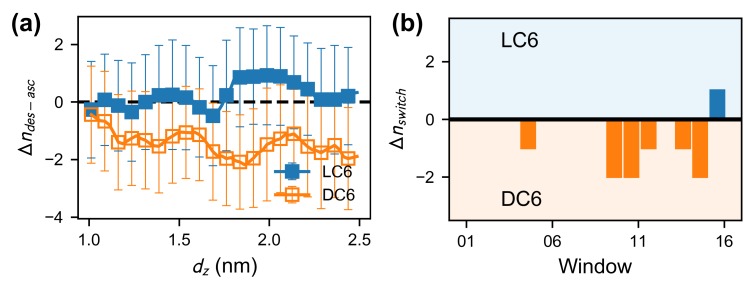
(**a**) The reweighted expectation and standard deviation of $\Delta n_{des-asc}$ (the difference of lipid numbers between the descending and ascending sides). The standard deviation is given only to show the fluctuation of $\Delta n_{des-asc}$, which reflects the flexibility of lipids. (**b**) The number difference ($\Delta n_{switch}=n_{switch}^{L}-n_{switch}^{D}$, where $n_{switch}^{X}$ is the number of lipids in the case of *X*C6 that experience switching between the des and asc sides during the simulations.) of lipids switching between descending and ascending sides.

By comparing the lipid distribution on the descending and ascending sides, it is observed that the lipid molecules may distribute nonuniformly around the peptide. The heterogeneity can be quantified as the difference of lipid numbers on the two sides as $\Delta n_{des-asc}=n_{des}-n_{asc}$, where $n_{des}$ ($n_{asc}$) is the number of ambient lipids on the descending (ascending) side. The expectation of $\Delta n_{des-asc}$ (Figure 5a) is calculated from the equilibrium probability distribution reweighted from the umbrella sampling. The quantities $\Delta n_{des-asc}$ for peptides LC6 and DC6 have different trends. For peptide LC6, the number difference $\Delta n_{des-asc}$ is almost 0 for all the locations. Differently, the difference $\Delta n_{des-asc}$ for the peptide DC6 is apparently smaller than 0, which indicates that there is a biased distribution of lipids around DC6, that is, fewer lipids gather on the descending side of the helix. This heterogeneity is consistent with the picture from the score $S_{rot}$. As the fewer lipids gathered on the descending side, fewer contacts (and weaker interactions) can be formed to drag the helix thus make the rotation difficult. The heterogeneity of lipids may accelerate lipid hopping between the two sides. This phenomenon is confirmed from the windows of umbrella sampling. It is found that there are much more lipids hopping between two sides of the DC6 helix (as shown in Figure 5b). The membrane looks more floppy in such a situation. This kind of hopping is more frequent in the windows 10–15, typically before the crossing of the free-energy barrier. This partly suggests that the nonuniform distribution is related to the free-energy barrier. As a comparison, the hopping lipids are less observed for peptide LC6, and most of the hopping events happen when the peptide is far away from the membrane. All these observations reflect that there are observable responses in the membrane, accompanied with the different rotations of peptide enantiomers, and the nonuniform distribution may act as a signature or a consequence of the slow activation processes.

### 2.6. The Key Role of Trp

Based on the above analysis, we find that the amino acid tryptophan is often located around the surface of the membrane, and interact with the lipid molecules. It is widely known that the tryptophan (Trp) enantiomers are most distinct from each other when adsorbed on the membrane [18]. This is partly related to the complex interactions of the heterocyclic ring in Trp [53,54,55]. The amino acid Trp is a good candidate to generate different behaviors when the chirality is under consideration. Will this residue also play important roles in the chirality-related peptide–membrane interaction? To further explore the microscopic mechanism of chirality-related behavior in the C6-membrane system, we come to study the interactions related to the tryptophan residue in peptide C6. Besides, in peptide C6, there is only one Trp. The orientation of the Trp side chain is synchronized with the helix orientation. The importance of rotational kinetics is another clue to attract our attention to study the interactions of Trp.

**Figure 6:**
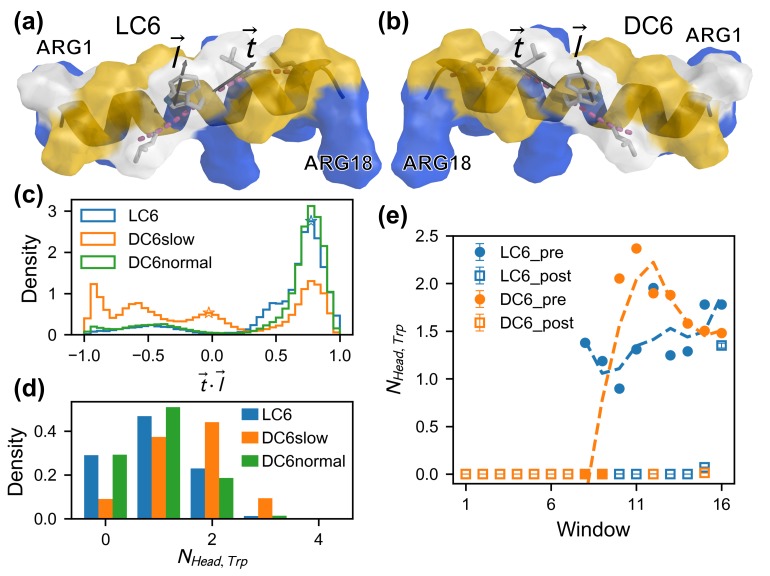
The diagram of surface helix fragments and the vectors employed to characterize the Trp indole orientation of LC6 (**a**) and DC6 (**b**). The four helix fragments are colored alternately in yellow and white. The diagrams are visualized with PyMOL [56]. (**c**) The distribution of the orientation of Trp indole measured as $\vec{t}\;\cdot \;\vec{l}$ for pre-adsorption states of kinetic trajectories. The pre-adsorption mean value for umbrella sampling windows of L/DC6 is marked as an unfilled star in corresponding color. (**d**) The distribution of contact number between Trp and lipid head groups for pre-adsorption states of kinetic trajectories. The means of the distributions in (**d**) are (in the order of LC6, DC6normal and DC6slow) 0.96, 0.92 and 1.54 with the sizes of the corresponding samples 50443, 70085 and 64324, respectively. (**e**) The averaged number of contacts between Trp and lipid head groups in the windows of umbrella sampling.

Visiting the helix structure in detail, we observe that the arrangement of nonpolar residues (Leu and Trp) in the helix effectively creates four stacked helix fragments on the peptide surface, as shown in Figure 6a,b. Trp is right on the longest fragment as denoted by the pink dashed lines in Figure 6a,b. it is observed that the lipids interacting with the nonpolar surface (on the descending side) prefer to be aligned with the grooves on the helices to maximize lipid–peptide contacts. Accompanied with the rotation of the peptide, sliding of the lipid tails along the grooves can be expected. Obviously, there are no penalties of free energy. Thus, This kind of processes would be in a downhill manner. This is always true for peptide LC6. Yet, for the peptide DC6, the side chain of the Trp has a probability to adopt other conformations. A flipping of Trp indole may happen, which disrupts the surface helices. This is observed in our simulations. This may destroy the interactions between the lipids and the hydrophobic surface of the peptide. Then, the rotation may be stuck. To illustrate such a kind of effect, we quantitatively characterize the flipping of Trp indole based on local geometry of helix cylinder. That is, the dot product $\vec{t}\;\cdot \;\vec{l}$ that measures the orientation of the Trp side chain in the frame defined based on local cylinder coordinate. Here, the vector $\vec{t}$ gives the direction of the local helix groove, and is calculated as the unit vector along the tangent line of the helix fragment that the residue Trp belongs to, and the vector $\vec{l}$ quantifies the direction of the Trp side chain, and is measured as the unit vector of the long axis of Trp indole (defined as the axis from CD1 to CH2), as shown in Figure 6a,b.

Based on the above analysis, the pre-adsorption state is more essential in the interaction between peptide and membrane. Thus, the distributions of side-chain orientation $\vec{t}\;\cdot \;\vec{l}$ are evaluated for the pre-adsorption state (as shown in Figure 6c). The data to calculate distributions are extracted from the kinetic trajectories. An average value of the orientation $\vec{t}\;\cdot \;\vec{l}$ for the pre-adsorption state is also calculated based on the data from umbrella sampling of L/DC6. The average values are marked as unfilled stars in Figure 6c. The results from two sources of data are consistent with each other. From Figure 6c, it is easy to observe that the distributions are not the same for various cases. For the case of peptide LC6 and of DC6 with normal kinetics (i.e., in the fast mode), the dot product $\vec{t}\;\cdot \;\vec{l}$ concentrates in a region from 0.6 to 0.9, which corresponds to a small angle between $\vec{t}$ and $\vec{l}$. That is, the vectors $\vec{t}$ and $\vec{l}$ are in a *cis* manner, and the direction of Trp indole is likely to be parallel with that of the groove. For the case of DC6 with slow kinetics, the orientation $\vec{t}\;\cdot \;\vec{l}$ distributes more uniformly. The peak around 0.8 is rather short, and there are several additional small peaks around 0, -0.6 and -1, respectively. Especially, there are significant distributions in the region ($\vec{t}\;\cdot \;\vec{l}\leq 0$). It indicates that two vectors are perpendicular to each other or in a *trans* manner. It is reasonable to conclude that the perpendicular and *trans* orientations are tightly related to the slow kinetics of adsorption. Phenomenologically, these orientations can be regarded as “bad-orientated”, which may block the groove and slow down the rotation. On the other hand, the *cis* orientation is “well-orientated”, making the rotation fluent. The relation between these two kinds of orientations and different kinetic effects demonstrates the importance of the residue Trp in C6 enantiomers.

It is more interesting to find out that there are direct interactions between the residue Trp and the polar head groups of lipids, though the residue Trp is located on the hydrophobic surface of the helix. This kind of interactions are defined as the contacts between Trp residue and head groups of lipid molecules. A Trp-lipid pair can form at most one contact. Thus, the number of contacts indicates the number of lipids which interact with the Trp residue. The statistics on the contacts between Trp and lipids are given in Figure 6d,e. It is found that there are quantitative differences between the interactions in various cases. Firstly, the average number of contacts for the cases of LC6 and of DC6 with normal kinetics is about 1 (0.96 and 0.92 exactly), and is roughly 2 (1.54 exactly) for the case of DC6 with slow kinetics. These results indicate that, in the slow kinetics, the residue Trp of the peptide DC6 is likely to bind with more lipid molecules (through their head groups). This kind of differences can also be observed during the adsorption process sampled in umbrella windows. The average numbers of contacts for the pre-adsorption and post-adsorption states are determined for various windows in umbrella sampling (as shown in Figure 6e). These describe the interaction between Trp and membrane in two typical kinds of states when the peptide is located at various depths. For the peptide DC6 with slow kinetics, the contact number increases when $d_{z}$ decreases from 2.5 nm–2.0 nm (corresponding to the window from 16–11), and then decreases rapidly when $d_{z}$ goes from 1.9 nm–1.7 nm (corresponding to the window 10–8). Meanwhile, the contact numbers for peptide LC6 are roughly constant in this region (namely, corresponding to the window from 16–8), and the values are generally smaller than those for the peptide DC6 with slow kinetics. Physically, the additional contacts represent the abnormal packing between Trp and lipid. With these additional contacts, the hydrophobic interface of helix would be deteriorated, which would impede the rotation of DC6 peptides.

The variation of the Trp-lipid contacts may help to explain many observations. In the region from $d_{z}=2.5$ to 2.0 nm (namely, in the window 16–11), the increase of additional Trp-lipid contacts indicates an increase of impedance effect. At the same time, the score $S_{rot}$ increases in this region. The concurrence of these two phenomena reflects a dynamic cancellation between the impedance (by the additional contacts between Trp and head lipid groups) and the driving force (from interaction differences between two sides of helix) during the rotation. Besides, in the region from 1.9 nm–1.7 nm (namely, from window 10–8), there is a rapid decrease of the Trp-lipid contacts, which may be the result from the recovery of this distortion. This region is in well accordance with the event of barrier crossing. It is worth pointing out that the “bad-orientation” of Trp indole and the preferential binding with lipid head groups could introduce disorder to the lipids interacting with the nonpolar surface (around the descending side). Hence, it is expected that fewer lipids would be recruited. This is well consistent with the observed heterogeneity in lipid distribution around the peptide DC6. Actually, the interactions between Trp and head groups of lipids are more important than other non-covalent interactions to produce chirality-related selection. If the whole lipid or only the tail group is used in the evaluation of contacts, the distributions of the contact number become flat and dispersed, and there is no systematic difference. The interaction between Trp and head group of lipid is the key ingredient in the peptide–membrane interaction.

As a conclusion, based on these analyses, an unusual orientation of the residue Trp and the preferential binding with lipid head groups are observed for the peptide DC6. These features are the source of the slow kinetics of the peptide DC6. It is clear that the residue Trp is the center to generate such kind of kinetics anomaly. Also based on the above analyses, these features are tightly related to the chirality of the peptide DC6. The residue Trp becomes the link to connect the structural chirality and the kinetic differences of C6 enantiomers. This is conceptually consistent with the chirality-dependent adsorption of monomeric Trp [18]. Indeed, this kind of asymmetry triggered by the chirality of the residue Trp is physically related to the chirality of lipid molecules. The polar interaction between Trp indole and the lipid head group is probably the physical origin. Together with the groove on the helix, the kinetic diversity can emerge as observed in the adsorption processes.

## 3. Materials and Methods

### 3.1. System Preparation

In our work, the CHARMM36 all-atom force field [57,58] is employed for both the peptide enantiomers and the membrane. Neutralizing ions are added to all systems. This kind of force field has been used in the simulations for -helical AMPs [49,52] and POPC membranes [59], and the results showed good agreements with the experiments, which implies the important balance of force field parameters for proteins and lipids [49]. All simulations are performed using GROMACS 5.1.4 package [60].

In our simulations, the initial conformation of the peptides are modeled as the ideal -helix, which is consistent with the experimental observations [28] as well as previous simulations on other AMPs [49,50,61]. To avoid biases on the interactions between peptides and the membrane, the helical peptides are initially placed parallel to the membrane surface, which is a common orientation for many AMPs in adsorption [62,63,64,65]. The Initial structures of the peptide–membrane systems are prepared by inserting the peptide into a pre-equilibrated POPC bilayer using the CHARMM-GUI website [66,67,68]. The lipids around the peptides are then equilibrated with stepwise-released position restraints on the peptides. Potentially there may be a risk that using a pre-equilibrated membrane configuration as initial setup for all trajectories may introduce artifacts. But due to the flexibility of lipids, this risk is negligible. All simulations are run at room temperature 303.15 K. The run parameters for vdW method, Coulomb method, barostat and thermostat are adopted according to the recommendation of the website. Simulations are performed with a length of 21.4 s in total.

### 3.2. Characterization of Helix Rotation

The helical peptide can be phenomenologically viewed as a heterogeneous cylinder (Figure 1a). The charged (polar) and hydrophobic residues distribute on different sides of the cylinder. Thus, the rotation around the axis of the cylinder would not be trivial and produce different states, especially with membrane as the spatial boundary. Practically, the side chain of residue Trp can be used to characterize the rotation of the peptide. The rotation angle θ is measured with the dihedral composed of the axis of the helix, the side chain of Trp (atom CG) and the positive direction of z-axis, as diagrammed in Figure 1b. Trp is picked as the indicator, because the indole group of the Trp residue is the largest hydrophobic side-chain among all residues of C6 peptide. This residue has strong and specific interactions with membrane [69,70], which may contribute significantly to the chirality-related interactions.

### 3.3. Adsorption of Monomeric Peptide

The concerned peptide is initially placed on the surface of a bilayer membrane with 128 POPC molecules (Figure 2b Pre). The following adsorption process is simulated by the MD simulation with semi-isotropic NPT ensemble. The typical span of the trajectory is 200 ns. Multiple trajectories (maximally 50) are simulated to gain a comprehensive picture about the kinetics of the adsorption.

### 3.4. Umbrella Sampling for the Adsorption Free Energy

Similar to many other simulations related to the peptide–membrane interaction, $d_{z}$, the distance between the COM of the peptide and that of the bilayer along the direction of membrane normal (defined as z-axis) is chosen as CV. In our simulations, $d_{z}$ ranges from 1.0 nm–2.5 nm. Along $d_{z}$, 16 sampling windows are generated uniformly. In each window, the initial conformation is generated from the typical kinetic trajectories, and a sampling over 200 ns is carried out after 100 ps equilibrium. Trajectories after 20 ns are employed to check convergence using the gmx wham tool. Based on these sampling results, the 2-dimensional (with peptide rotation θ as the second CV) PMF profiles are calculated using the multistate Bennett acceptance ratio estimator (MBAR) [71]. The reweighted PMFs from wham and MBAR show good consistency. The trajectories from each window are further analysed as described in the next subsection.

### 3.5. Analysis on Umbrella Windows

Analysis on window trajectories is performed to investigate the underlying details of the interactions. The investigation is concentrated on local interactions between the peptide and ambient lipids, which are filtered as any lipid that resides within 3 *Å* of the peptide. The lipid–peptide distance is determined by the nearest atoms from the two molecules. The ambient lipids are further classified into two groups: group on the descending side and on the ascending side, according to the side on which the chiral carbon, i.e., atom C2 is located. Classification based on COM of lipids are also performed. The results are similar to the C2-based analyses. According to the rotation angle, states pre-adsorption ($\theta >225{}^{\circ }$ for LC6 and $\theta <135{}^{\circ }$ for DC6) and post-adsorption ($\theta <180{}^{\circ }$ for LC6 and $\theta >180{}^{\circ }$ for DC6) are characterized to compare the microscopic differences. Contacts between protein residues and lipids are calculated based on a criterion of 5 *Å* for the distances between heavy atoms calculated with the python package MDTraj [72]. The contacts are then classified according to the hydrophobicity of residues and the location of lipids as aforementioned. The number of contacts are calculated for all classes and are summed with the weights described in the Supplementary Materials.

## 4. Conclusions

In this work, we try to answer the question regarding how the chirality of peptides produces different kinetics. Based on molecular dynamics simulations, we observed kinetic differences for the adsorption of C6 enantiomers, which is qualitatively consistent with experiments. Through the analysis of the energy landscape, rotational motion of the helix and molecular interactions during the kinetic processes, we conclude that the difference in interactions between residue Trp of C6 enantiomers and the chiral lipid molecules are microscopic reasons for the kinetic asymmetry. These results demonstrate that, around a certain interface (here, the groove interface of peptide helix), the chirality-related interaction may emerge due to geometric restrictions. A similar thing happens for the adsorption of the amino acid Trp on a membrane surface [18]. Moreover, the side chain of the residue Trp is bifacial to interact with the lipid, being hydrophobic with the lipid tail and being polar with the lipid head. This characteristic of interaction may constrain the local conformations of Trp and lipid, so that the chirality may play its role in the molecular interactions. This might be another requirement for chirality-dependent behavior. We believe that this information may help to understand when and how the chirality contributes to molecular kinetics, and may shed light on the chiral molecular biophysics and stimulate the design of functional molecules that benefit from chirality-dependent effects.

## Supplementary Materials

The following are available online at https://www.mdpi.com/1422-0067/20/19/4760/s1, Table S1: Weights for contact summation, Figure S1: Diagram of the rotation process of helix, Figure S2: Secondary structure records of typical monomeric trajectories, Figure S3: $S_{CD}$ order parameters of peptide-surrounding lipids. Figure S4: Histograms within the umbrella sampling windows. Figure S5: Equilibrium probability dstribution of $\Delta n_{des-asc}$.

## Abbreviations

The following abbreviations are used in this manuscript:

AMP	antimicrobial peptide
LC6	the C6 peptide composed of L-type amino acids
DC6	the C6 peptide composed of D-type amino acids
COM	center of mass
CCD	complementary cumulative distribution
FPT	first passage time
PMF	potential of mean force
CV	collective variable
SEM	standard error of the mean
MBAR	multistate Bennett acceptance ratio estimator
