# Supplementary material for: Chirality-Dependent Adsorption between Amphipathic Peptide and POPC Membrane
Source: Int J Mol Sci. 2019 Sep 25;20(19):4760. doi: 10.3390/ijms20194760 (PMC6801444; doi:10.3390/ijms20194760)
Supplement: Supplementary file 1 [file ijms-20-04760-s001.pdf]

Article

Supplementary Materials: Chirality Dependent Adsorption between Amphipathic Peptide and POPC Membrane

Ke Chen [1], Yuebiao Sheng [1]*, Jun Wang [1]* and Wei Wang [1]*

[1] National Laboratory of Solid State Microstructure, Collaborative Innovation Center of Advanced Microstructures, and School of Physics, Nanjing University, Nanjing 210093, P.R. China

* Correspondence: syb@nju.edu.cn (Y. S.); wangj@nju.edu.cn (J. W.); wangwei@nju.edu.cn (W. W.)

Version September 11, 2019 submitted to Int. J. Mol. Sci.

Keywords: chirality; protein; membrane; POPC; tryptophan; molecular dynamics

1. Determination of the weights for summation of contacts

Table S1. Weights for contact summation

θ (**L**, °)	θ (**D**, °)	w_{dp}	w_{dh}	w_{ah}	w_{ap}
360–310	0–50	−1	0.5	−1	0.5
310–270	50–90	−0.5	1	−1	0.5
270–220	90–140	−0.5	1	−0.5	1
220–180	140–180	0	0	0.5	0.5
180–130	180–230	1	−0.5	1	−0.5
130–90	230–270	1	−0.5	0.5	−1.0

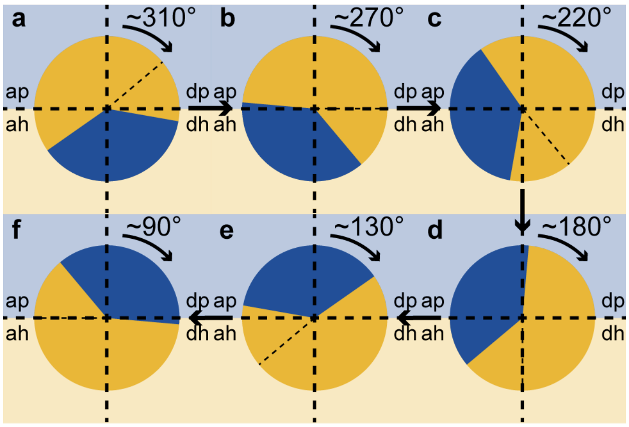

Figure S1. Diagram of the rotation process from the cylindrical section of C6 helix at $\theta = 310°$ (**a**), 270° (**b**), 220° (**c**), 180° (**d**), 130° (**e**) and 90° (**f**). The side chain of Trp, e.g. the indicator of θ, is diagrammed by the thin dashed line on the circle.

The polar angle of C6 can be determined as 140°. The diagram of amphipathic heterogeneity is based on the polar angle. Note the amphipathic feature of peptide is actually resulted from the sidechains, hence the amphipathic surface is not fully rigid. The rotation states are diagrammed with approximate θ values.

In membrane environment, the ambience of helix can be split into quarters of dp (descending-polar), dh (descending-hydrophobic), ah (ascending-hydrophobic) and ap (ascending-polar). These quarters are considered only to form contacts with the helix surface of the same hydrophobic/polar type. The positive/negative contribution of each quarter is related to increasing/decreasing of the area of corresponding polar/hydrophobic surface in corresponding motion side. Each quarter is thought to fully contribute to the change of corresponding area in the local quarter, and half contribute to the change of corresponding area in the neighboring quarter (the quarter sharing the same motion side with local quarter). Hence the weights for local are given as 1 if area of the corresponding surface in local quarter increases during the rotation, else -1; the weights for neighbor is 1/2 if area of the corresponding surface in the neighboring quarter increases, else -1/2. The overall weight for the concerned quarter is the summation of weights for local and neighbor quarters.

2. Secondary Structure Records During the Adsorption of C6 Enantiomers

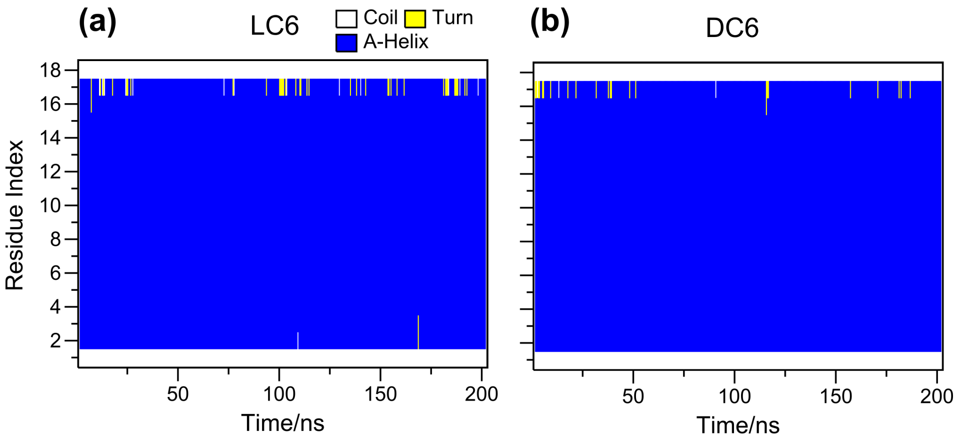

Figure S2. Secondary structure records of typical monomeric trajectories of LC6 (**a**) and DC6 (**b**). The secondary structure is determined by DSSP with the gmx do_dssp tool.

In all our 107 trajectories (25 for monomeric LC6, 50 for monomeric DC6 and 16 windows for each enantiomer), the helix structure is well preserved with only occasionally unfolded terminal residues. The middle region of the helix (the location of the Trp residue) is in good helix conformation at all times and no significant bending (as melittin) is observed.

3. S_{CD} Order Parameters of Peptide-Surrounding Lipids

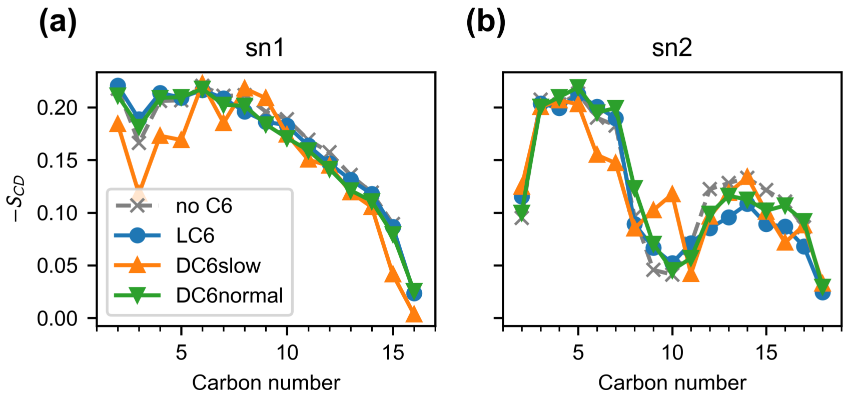

Figure S3. The S_{CD} order parameters of the lipids within 3 Å of the peptide. The parameters for chain sn1 (**a**) and sn2 (**b**) in the monomeric simulations are shown. Chain sn1 is saturated and chain sn2 is monounsaturated at carbon 9. The parameters calculated from a POPC membrane without peptide (labeled as no C6) is plotted as reference.

Generally the S_{CD} of peptide-surrounding lipids are similar to the pure POPC membrane that without peptides, with relatively larger deviations on certain carbon atoms in the case of the slow mode of DC6. The S_{CD} data suggests that the peptide-surrounding lipids preserve similar flexibility as in the bulk membrane, though the special interaction in the slow mode of DC6 may indeed cause some distortion on the behavior of lipids.

4. Histograms within the Umbrella Sampling Windows

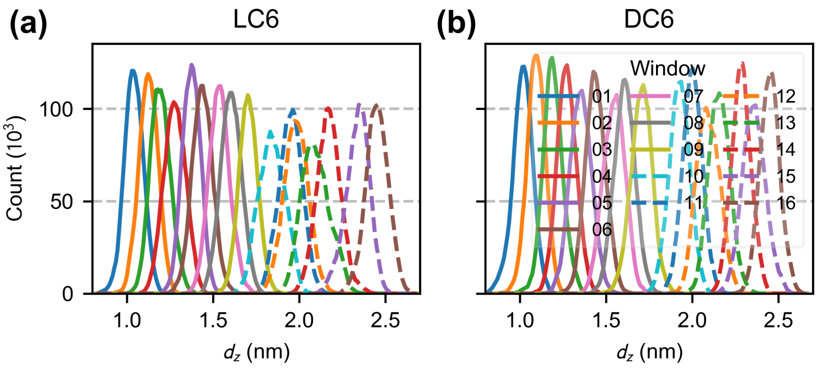

Figure S4. The histograms of CV (d_z) within the umbrella sampling windows for LC6 (**a**) and DC6 (**b**).

The histograms of CV shows that there is sufficient overlap between adjacent windows for both LC6 and DC6.

5. Equilibrium Probability Distribution of $\Delta n_{des-asc}$

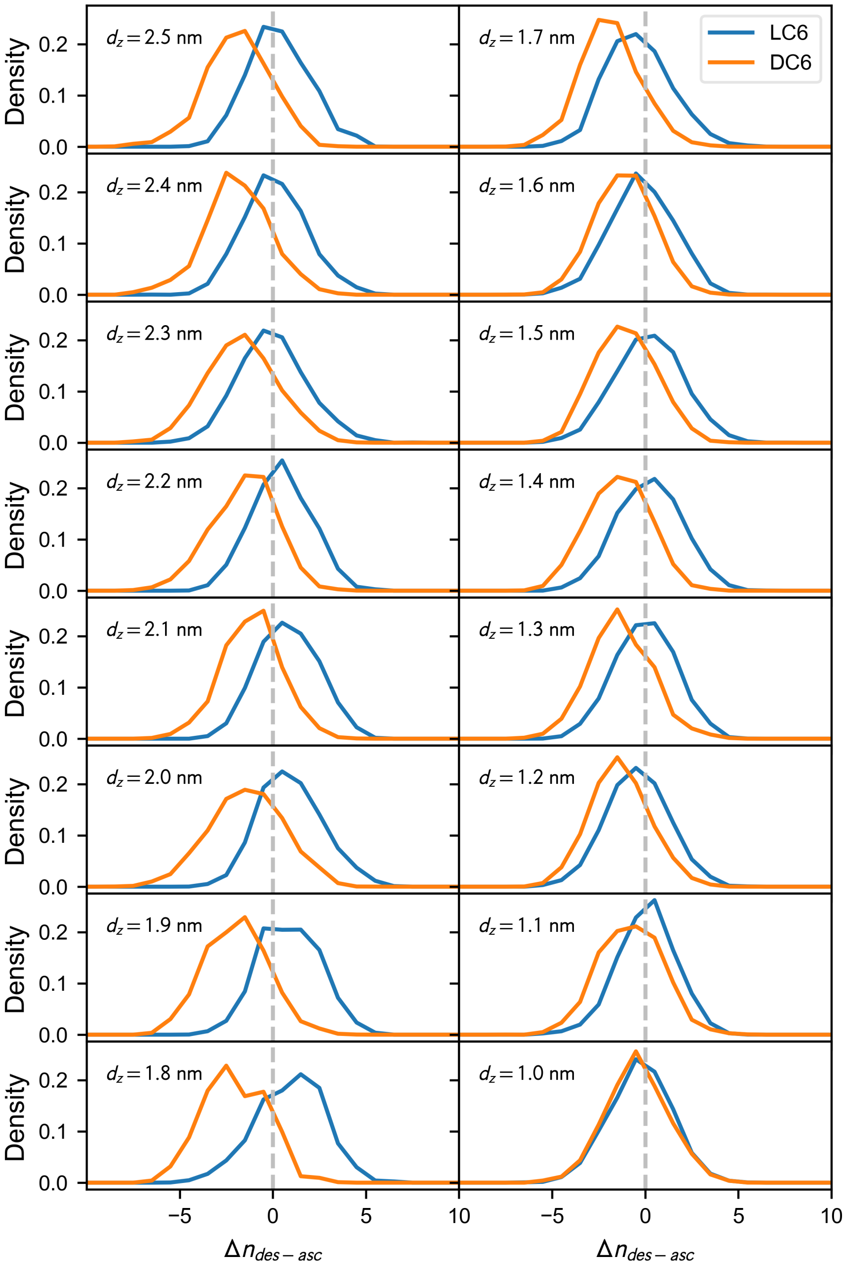

Figure S5. The equilibrium probability distribution of $\Delta n_{des-asc}$ for LC6 and DC6 from $d_z = 2.5$ to 1.0 nm. In most cases, especially when before the barrier-crossing (the left column with $d_z \geq 1.8$ nm), the distributions from the C6 enantiomers can be clearly distinguished from each other.

© 2019 by the authors. Submitted to *Int. J. Mol. Sci.* for possible open access publication under the terms and conditions of the Creative Commons Attribution (CC BY) license (http://creativecommons.org/licenses/by/4.0/).
